# Robotic evolution from Si to Xi in rectal cancer assessing operative performance and oncological outcomes

**DOI:** 10.3389/fsurg.2025.1668213

**Published:** 2026-01-21

**Authors:** Wenpeng Wang, Shan Gao, Jinghao Huang, Duo Yun, Jiefu Wang

**Affiliations:** 1 Department of Colorectal Oncology, Tianjin Medical University Cancer Institute and Hospital, National Clinical Research Center for Cancer, Tianjin’s Clinical Research Center for Cancer, Tianjin Key Laboratory of Digestive Cancer, Tianjin, China; 2 Department of Immunology, Tianjin Medical University Cancer Institute and Hospital, National Clinical Research Center for Cancer, Key Laboratory of Cancer Prevention and Therapy, Tianjin's Clinical Research Center for Cancer, Tianjin, China; 3 Division of Hepatobiliary and Pancreas Surgery, Department of General Surgery, Shenzhen People’s Hospital (the Second Clinical Medical College, Jinan University; The First Affiliated Hospital, Southern University of Science and Technology), Shenzhen, China; 4 Cancer Center, Beijing Friendship Hospital, Capital Medical University, Beijing, China

**Keywords:** postoperative complications, prognosis, rectal neoplasms, robotic surgical procedures, robotics

## Abstract

**Purpose:**

To compare perioperative and oncologic outcomes between robotic surgical platforms (Si vs. Xi) in rectal carcinoma.

**Methods:**

A retrospective cohort study of 86 robotic rectal cancer resections (Si: *n* = 31; Xi: *n* = 55) were analyzed at Tianjin Medical University Cancer Hospital between November 2019 and June 2024.

**Results:**

Among 86 patients with comparable baseline clinicopathological variables (all *p* > 0.05), the Xi system showed superior perioperative efficiency: shorter operation (226.7 vs. 282.1 min, *p* = 0.010), console (*p* = 0.016) and docking times (*p* = 0.013), less blood loss (83.8 vs. 155.8 mL, *p* = 0.005), and a shorter postoperative stay (7.8 vs. 9.7 days, *p* = 0.016). On multivariable analyses, Xi remained independently associated with a shorter operative time (*p* = 0.002), reduced blood loss (*p* = 0.027), and decreased length of stay (*p* = 0.038). Complication rates, lymph node yield, and short-term oncologic quality indicators (distal resection margin [DRM], circumferential resection margin [CRM], mesorectal integrity) were comparable between two systems (all *p* > 0.05). In low rectal cancers (≤ 5 cm from the anal verge) with balanced baselines, Xi achieved a higher sphincter preservation rate (90.5% vs. 55.6%, *p* = 0.049). Survival trends numerically favored Xi (3-year DFS 79.8% vs. 73.0%; OS 92.0% vs. 83.0%), but differences were not significant (DFS: *p* = 0.54; OS: *p* = 0.26). On Cox regression, TNM stage independently predicted both DFS (*p* = 0.041) and OS (*p* = 0.029). However, the robotic platform (Xi vs. Si) showed no survival advantage (DFS: HR = 1.33, 95% CI 0.53–3.37, *p* = 0.548; OS: HR = 1.43, 95% CI 0.76–2.67, *p* = 0.267).

**Conclusions:**

Compared with Si, the Xi platform confers measurable perioperative advantages—shorter operative time, less blood loss, and reduced hospitalization—without compromising short-term oncologic quality or survival. In low rectal tumors, Xi may facilitate sphincter preservation under comparable baselines. Long-term outcomes appear driven primarily by disease stage rather than platform generation.

## Introduction

The evolution of robotic-assisted surgical systems has revolutionized minimally invasive approaches in rectal cancer surgery (1, 2). Robotic-assisted surgery surpasses standard laparoscopy through improved intracorporeal maneuverability, enhanced stereoscopic visualization, motion-filtered precision, reduced operator fatigue (3). The da Vinci Surgical System, with its successive generations Si and Xi, has been instrumental in improving surgical precision and patient outcomes in rectal cancer treatments, particularly in surgeries performed in narrow spaces that fully leverage the advantages of a surgical robotic system (4). The fourth-generation da Vinci Xi system offers several improvements over its predecessor, including enhanced operational flexibility and efficiency, especially in complex multiquadrant surgeries (5).

The current evidence base from pivotal randomized controlled trials (RCT) has primarily contrasted robotic-assisted proctectomy with laparoscopic approaches rather than evaluating inter-generational robotic systems. The seminal ROLARR trial, while demonstrating comparable conversion rates between robotic-assisted proctectomy (RAP) and laparoscopic proctectomy (LP) overall, revealed critical robotic advantages in technically challenging subgroups—particularly in male patients and low rectal tumors (6). The COLRAR trial showed RAP achieved better CRM negativity (100% vs. 93.9%) in neoadjuvant-treated patients (7). While proving robotic surgery's value, it didn't compare different robot models. The later REAL trial—the first large multi-center study comparing RAP and LP with long-term results—showed that RAP worked better than LP, with fewer cancer recurrences after 3 years. However, like previous studies, it didn't compare different versions of the robotic system (8).

In our prior study comparing robotic-assisted (RACS) and laparoscopic colorectal surgery (LCS), we demonstrated that RACS, despite superior visualization, involved longer operative times and greater blood loss than LCS, with comparable clinical outcomes (9). However, that study focused on broad comparisons between robotic and laparoscopic approaches across colorectal cancer (CRC) subtypes, without evaluating technical differences between specific robotic platforms.

While previous studies have explored the benefits of robotic systems in enhancing dexterity and reducing invasiveness, direct comparisons between Si and Xi systems concerning postoperative complications and long-term prognosis in rectal cancer surgery remain limited (10, 11). The current study extends our previous work by specifically analyzing the evolution from Si to Xi platforms, with a focus on rectal cancer. Here, we assess novel metrics such as sphincter preservation rates in low tumors (≤5 cm from the anal verge), docking time efficiency, and platform-specific oncological outcomes-critical parameters that were not assessed in our prior investigations. This study demonstrates how improvements from older to newer robotic systems enhance both short-term surgical results and long-term survival for challenging rectal cancer case.

## Methods

### Patient selection

A cohort of 86 patients with rectal cancer who underwent robotic-assisted surgery (Intuitive Surgical, Inc., USA) between November 2019 and June 2024 were retrospectively enrolled and analyzed. Our institution had been equipped with the third-generation da Vinci Si system since 2016, and the fourth-generation da Vinci Xi system was introduced in 2019. Before the inclusion of patients in this study, our colorectal surgical team underwent structured training and proficiency certification to ensure technical consistency across both systems. The Si system was employed between November 2019 and December 2020, and beginning in January 2021, both Si and Xi platforms were concurrently available for rectal cancer surgery. Therefore, patients in the Si group were operated slightly earlier on average. However, baseline demographic and tumor characteristics, as well as surgeon experience level, were comparable between groups, minimizing potential chronological bias. In addition, the choice of robotic system after 2021 primarily depended on system availability and operating room scheduling, rather than on patient characteristics or surgeon preference.

Inclusion criteria were as follows: (1) Histologically confirmed adenocarcinoma of the rectum; (2) Clinically staged M0 disease or resectable M1 metastases with intestinal obstruction amenable to minimally invasive surgery; (3) Candidate for elective curative-intent resection; (4) Willingness to undergo robotic approach.

Exclusion criteria included incomplete data, severe comorbidities (defined as American Society of Anesthesiologists [ASA] class ≥ III, including conditions such as advanced heart failure [NYHA class III/IV], uncontrolled diabetes [HbA1c > 9% with end-organ damage], severe COPD [GOLD stage 3–4], or end-stage renal disease on dialysis), history of prior related treatments or surgeries, inadequate follow-up, poor treatment adherence, or ethical non-compliance. Ethical approval was obtained from the Institutional Review Board (IRB) of Tianjin Medical University Cancer Institute Hospital (No. bc20240922).

### Surgical team

To minimize potential bias and variability, all procedures were performed by the same experienced surgical team across both Si and Xi groups. Prior to the study, our team had completed ≥30 robotic cases—surpassing the initial learning curve—and demonstrated proficiency by performing 15 robotic rectal cancer surgeries within 90 days post-training. This consistency ensures that differences in outcomes primarily reflect the robotic systems' capabilities rather than disparities in surgical expertise.

### Surgical approach and procedure

**Figure 1:**
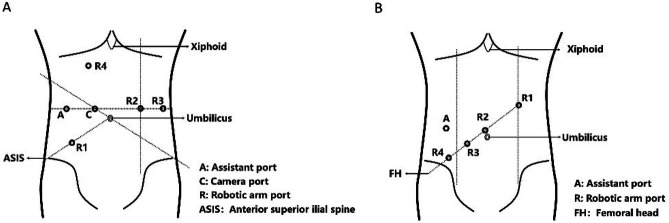
The figure illustrates two different configurations for port placement in robotic-assisted surgery. **A**, Si; **B**, Xi.

The trocar placement for da Vinci Si robotic rectal resection surgery was as follows: A 12 mm camera port (C) was placed 3–4 cm above the umbilicus to the right. An 8 mm robotic arm port (R1) was positioned at McBurney's point, located at the lateral one-third of the line between the umbilicus and the right anterior superior iliac spine. Another 8 mm robotic arm port (R2) was placed on the left midclavicular line at the same level as the camera port. An 8 mm robotic arm port (R3) was positioned on the left anterior axillary line, also level with the camera port, and was primarily used for assisting with low rectal dissection. For splenic flexure mobilization, an 8 mm robotic arm port (R4) was placed 3–4 cm below the xiphoid process, midway between the midline and the right midclavicular line. Additionally, a 5 mm/12 mm assistant port (A) was positioned on a vertical line through R1 and level with the camera port. Ports R1, R2, and/or R3 were utilized for mobilizing the rectum and sigmoid colon, while ports R1, R4, and/or R3 were used for mobilizing the splenic flexure (Figure 1A).

For rectal resection using the da Vinci Xi robotic system, Port 1 (R1) was positioned on the midline at the left subcostal margin for initial insufflation and could serve as the camera port. Port 2 (R2), primarily used for the camera, was placed at the intersection of the midline and a line drawn from the right femoral head to the left midclavicular line (MCL). Ports 3 and 4 (R3 and R4), spaced 8 cm apart along this line, were used for instrument arms. The assistant port (A) was triangulated away from the robotic arms, lateral to the right MCL. Port placements were adjusted based on patient anatomy, with a spacing of 6–10 cm between robotic ports, and dual docking was utilized for extended surgical fields (Figure 1B).

### Definition of variables and procedure terminology

Operation time: From the first incision to completion of surgical suturing. Intraoperative blood loss: Total blood volume lost during the procedure. Lymph node yield: Number of lymph nodes harvested. Postoperative hospital stay: Days from surgery completion to discharge. Postoperative complications included anastomotic leak (bowel anastomosis disruption with enteric leakage), abdominal infection (imaging/surgery-confirmed abscess/peritonitis), urinary tract infection (positive urine culture with symptoms), bleeding (Hb drop >2 g/dL requiring intervention), and perineal infection (purulent wound discharge with positive culture).

### Short- and long-term oncological outcomes

All patients underwent either Dixon (anterior resection) or Miles (abdominoperineal resection) surgery, both incorporating total mesorectal excision (TME). Pathological assessment of TME quality included: (1) distal resection margin (DRM; tumor-free distance ≥1 cm); (2) circumferential resection margin (CRM; positive if tumor ≤1 mm from the margin); (3) mesorectal integrity (graded as complete [intact], near-complete [minor defects], or incomplete [major defects] per Nagtegaal criteria) (12); (4) extramural vascular invasion (EMVI; tumor in vessels beyond muscularis propria); and (5) perineural invasion (PNI; tumor along nerve sheaths).

Patients were followed until June 2024 (median 37 months, range 3–59). Follow-up time was measured from surgery to the event or censoring; patients without events were censored at their last clinical contact, and an identical administrative cut-off was applied to both Si and Xi groups. Postoperative surveillance included quarterly visits for 2 years, semiannual visits during years 3–5, and annual visits thereafter. Disease-free survival (DFS) was defined as time from surgery to radiologically confirmed recurrence (RECIST 1.1) or distant metastasis (dual-imaging/histopathology verified). Overall survival (OS) spanned surgery to death.

### Statistical analysis

Data were analyzed using R 3.4.2 and SPSS 27.0 (IBM Corp., USA). For each continuous variable, normality was assessed with the Shapiro–Wilk test (plus Q–Q plots) and variance homogeneity with Levene's test. Variables meeting normality—operative time, console time, docking time, postoperative hospital stay, lymph node yield, and time to first flatus—were compared using two-sample *t*-tests (or Welch's t when variances were unequal). Blood loss exhibited right-skewness and was compared using the Wilcoxon rank-sum test. All continuous variables are summarized as mean ± standard deviation (SD) for consistency. Categorical variables were compared with *χ*^2^ or Fisher's exact tests. Beyond univariate tests, operative time, intraoperative blood loss, and postoperative hospital stay were analyzed using multivariable linear regression with adjustment for platform, age, sex, body mass index (BMI), T category, lymph node yield, neoadjuvant therapy, and surgical approach. Survival was evaluated by Kaplan–Meier curves and log-rank tests. DFS/OS were analyzed using univariate and multivariable Cox proportional hazards models adjusting for platform, TNM stage, tumor deposit, neoadjuvant therapy, age, and sex. Statistical significance was set at *p* < 0.05.

### Power/detectable-effect considerations

We performed a *post-hoc* power/detectable-effect analysis to quantify study resolution (two-sided *α* = 0.05; Si: *n* = 31, Xi: *n* = 55). For continuous outcomes, the minimal detectable standardized mean difference at 80% power is approximately Cohen's d ≈ 0.60 (i.e., powered for moderate effects). Using the observed means and pooled SDs, standardized between-group differences were d = 0.83 for operative time (pooled SD ≈ 66.6 min), d = 0.79 for console time (≈ 64.6 min), d = 0.78 for docking time (≈ 3.77 min), d = 0.79 for blood loss (≈ 91.6 mL), and d = 0.74 for postoperative length of stay (≈ 2.55 days), each corresponding to >90% approximate power with the current sample size. In contrast, lymph node yield (d ≈ 0.24) and time to passage of flatus (d ≈ 0.15) were underpowered to detect small differences. For time-to-event endpoints, power depends on the number of events; using Schoenfeld's approximation, the 80%-power detectable hazard ratio (HR) is $HR\approx \exp\left\{\pm \sqrt{{\left(1.96+0.84\right)}^{2}/E}\right\}$. In our cohort, DFS had 18 events (detectable HR ≈ 0.52 or 1.93), and OS had 12 events (detectable HR ≈ 0.45 or 2.24), indicating adequate power for moderate differences in DFS but limited power for small differences in OS.

## Results

### Patient characteristics

Table 1 presents the clinical and pathological characteristics of 86 patients who underwent surgery using the da Vinci Si and Xi systems. The da Vinci Si group included 31 patients, while the Xi group comprised 55 patients. There were no significant differences between the two groups in terms of age, sex, BMI, or histopathological parameters, including degree of histological differentiation, tumor type, presence of tumor deposits, or AJCC TNM stage.

**Table 1:** Patient baseline characteristics and pathological outcomes.

Variable	Si (*n* = 31)	Xi (*n* = 55)	*P*
Age (year-old)			0.671
≤61	16 (51.6%)	31 (56.4%)	
>61	15 (48.4%)	24 (43.6%)	
Sex			0.476
Female	15 (48.4%)	31 (56.4%)	
Male	16 (51.6%)	24 (43.6%)	
BMI (kg/m^2^)			0.415
<18.5	2 (6.5%)	1 (1.8%)	
18.5–24.9	23 (74.2%)	39 (70.9%)	
>25	6 (19.4%)	15 (27.3%)	
Differentiation, *n* (%)			0.171
Low	1 (3.2%)	9 (16.4%)	
Middle	28 (90.3%)	44 (80.0%)	
Unspecified	2 (6.5%)	2 (3.6%)	
Histology			0.928
Adenocarcinoma	28 (90.3%)	50 (90.9%)	
Mixed adenocarcinoma^a^	3 (9.7%)	5 (9.1%)	
T stage			0.307
T1	5 (16.1%)	3 (5.5%)	
T2	10 (32.3%)	15 (27.3%)	
T3	13 (41.9%)	32 (58.2%)	
T4	3 (9.7%)	5 (9.1%)	
N stage			0.374
N0	20 (64.5%)	34 (61.8%)	
N1	9 (29.0%)	12 (21.8%)	
N2	2 (6.5%)	9 (16.4%)	
M stage			0.283
M0	31 (100.0%)	53 (96.4%)	
M1	0 (0%)	2 (3.6%)	
TNM stage			0.397
I	12 (38.7%)	14 (25.5%)	
II	8 (25.8%)	20 (36.4%)	
III	11 (35.5%)	19 (34.5%)	
IV	0 (0%)	2 (3.6%)	
Tumor deposit			0.405
Negative	27 (87.1%)	44 (80.0%)	
Positive	4 (12.9%)	11 (20.0%)	
Neoadjuvant therapy			0.171
No	25 (80.6%)	50 (90.9%)	
Yes	6 (19.4%)	5 (9.1%)	
Surgery approach			0.460
Dixon	28 (90.3%)	52 (94.5%)	
Miles	3 (9.7%)	3 (5.5%)	
Distance from the anal verge (cm)			0.393
>5	22 (71.0%)	34 (61.8%)	
≤5	9 (29.0)	21 (38.2%)	

BMI, body mass index.

a Mixed adenocarcinoma including mucinous adenocarcinoma, signet ring cell carcinoma and other pathological types.

Pathologic evaluation revealed 78 cases of adenocarcinoma and 8 cases of mixed adenocarcinoma, which included mucinous adenocarcinoma, signet-ring cell carcinoma, and other subtypes. Two patients with M1 stage disease and liver metastases underwent surgery or radiofrequency ablation. Eleven patients with mid-to-low rectal cancer staged at T3 or higher, or with regional lymph node involvement, received neoadjuvant therapy before surgery. These patients demonstrated either partial response or stable disease following therapy. Surgical approaches included Dixon and Miles procedures, with no significant differences between two groups. There was no significant difference in tumor distance from the anal verge between two groups (*p* = 0.393).

Importantly, there were no perioperative mortalities in either group. The distribution of clinicopathological characteristics was comparable between the Si and Xi groups (all *p* > 0.05). This indicates that both systems provided similar surgical outcomes in terms of patient characteristics and tumor profiles.

### Perioperative surgical details

Mean operative time, console time and docking time were less in the Xi group than the Si group (*p* < 0.05 for all). Moreover, there was significantly more blood loss in the Si group than in the Xi group (mean, 155.81 mL vs. 83.82 mL, *p* < 0.001). The length of postoperative hospital stay in the Xi group was less than that in the Si group, and there was a significant difference (mean, 7.78 days vs. 9.68 days, *p* < 0.05). No significant difference was observed in the total number of lymph nodes yield in Si and Xi (mean, 15.35 vs. 16.84, *p* = 0.171). In addition, there were no significant differences observed between the two groups in time to passage of flatus (mean for Si and Xi, 3.68 days vs. 3.58 days, *p* = 0.658; Table 2).

**Table 2:** Perioperative surgical details.

Variable	Si [mean (SD)]	Xi [mean (SD)]	*P*
Operation time (min)	282.13 ± 80.76	226.71 ± 57.23	0.010
Console time (min)	249.23 ± 77.37	198.42 ± 56.33	0.016
Docking time (min)	11.74 ± 4.46	8.78 ± 3.33	0.013
Blood loss (mL)	155.81 ± 142.75	83.82 ± 41.48	0.005
Lymph node yield	15.35 ± 8.09	16.84 ± 4.89	0.171
Time to passage of flatus (days)	3.68 ± 0.65	3.58 ± 0.71	0.658
Postoperative hospital stay (days)	9.68 ± 3.62	7.78 ± 1.69	0.016

Data are presented as mean ± SD, SD, standard deviation.

In the multivariable linear regression for operative time, use of the da Vinci Xi platform (vs. Si) was independently associated with a shorter operative duration (*p* = 0.002). Male (*p* = 0.014), neoadjuvant therapy (*p* = 0.012), and Miles surgical approach (*p* = 0.045) were each associated with longer operative time. BMI showed a positive trend without reaching significance (*p* = 0.077). Age, pathological T category (T2–T4 vs. T1), lymph node yield, and intraoperative blood loss were not significantly associated with operative time (all *p* > 0.05; Table 3).

**Table 3:** Multivariable linear regression for operative time in robotic rectal surgery.

Variable	B	SE	*β*	t	*P*	95% CI	
da Vinci system (Si )	−47.03	14.63	−0.32	−3.22	0.002	−76.18	−17.88
Age (year-old)	−1.14	0.75	−0.14	−1.51	0.135	−2.64	0.36
Sex (Female )	34.03	13.50	0.24	2.52	0.014	7.14	60.92
BMI (kg/m2)	4.07	2.27	0.18	1.79	0.077	−0.45	8.59
T2 [ref (T1)]	9.88	25.79	0.06	0.38	0.703	−41.51	61.28
T3 [ref (T1)]	10.80	24.19	0.08	0.45	0.657	−37.40	59.00
T4 [ref (T1)]	11.24	30.75	0.05	0.37	0.716	−50.02	72.50
Lymph node yield	0.28	1.16	0.03	0.25	0.807	−2.02	2.58
Neoadjuvant therapy [ref (No)]	53.86	20.89	0.25	2.58	0.012	12.23	95.49
Surgery approach [ref (Dixon)]	56.60	27.75	0.20	2.04	0.045	1.31	111.89
Blood loss (mL)	0.11	0.09	0.15	1.24	0.220	−0.07	0.28

B, unstandardized regression coefficient; SE, standard error; β, standardized regression coefficient; CI, confidence interval; BMI, body mass index.

As detailed in Table 4, the multivariable model for intraoperative blood loss identified the surgical platform and approach as independent predictors. Use of the da Vinci Xi system was associated with significantly reduced blood loss (*p* = 0.027), while the Miles approach predicted increased blood loss (*p* = 0.004). Multivariable linear regression identified the robotic surgical platform as the only factor independently associated with postoperative hospital stay (Table 5). The use of the da Vinci Xi system was significantly associated with a shorter hospital stay compared to the Si system (*p* = 0.038).

**Table 4:** Multivariable analysis of factors associated with blood loss in patients undergoing robotic rectal surgery.

Variable	B	SE	β	t	*P*	95% CI	
da Vinci system (Si )	−45.53	20.21	−0.23	−2.25	0.027	−85.80	−5.26
Age (year-old)	1.21	1.01	0.11	1.20	0.235	−0.81	3.23
Sex (Female )	31.04	18.46	0.16	1.68	0.097	−5.75	67.82
BMI (kg/m2)	−4.58	3.05	−0.15	−1.50	0.138	−10.66	1.51
T2 [ref (T1)]	−3.99	34.53	−0.02	−0.12	0.908	−72.79	64.82
T3 [ref (T1)]	−16.42	32.34	−0.09	−0.51	0.613	−80.87	48.02
T4 [ref (T1)]	22.48	41.08	0.07	0.55	0.586	−59.38	104.33
Lymph node yield	−2.17	1.52	−0.14	−1.43	0.158	−5.21	0.87
Neoadjuvant therapy [ref (No)]	47.06	28.66	0.16	1.64	0.105	−10.04	104.15
Surgery approach [ref (Dixon)]	106.94	36.06	0.28	2.97	0.004	35.08	178.80
Operative time (min)	0.19	0.15	0.14	1.24	0.220	−0.12	0.50

B, unstandardized regression coefficient; SE, standard error; β, standardized regression coefficient; CI, confidence interval; BMI, body mass index.

**Table 5:** Factors independently associated with postoperative hospital stay after robotic rectal surgery.

Variable	B	SE	β	t	*P*	95% CI	
da Vinci system (Si )	−1.34	0.63	−0.24	−2.11	0.038	−2.60	−0.08
Age (year-old)	−0.02	0.03	−0.05	−0.48	0.633	−0.08	0.05
Sex (Female )	0.92	0.57	0.17	1.61	0.111	−0.22	2.06
BMI (kg/m2)	0.01	0.09	0.01	0.12	0.907	−0.18	0.20
T2 [ref (T1)]	0.63	1.05	0.11	0.60	0.551	−1.46	2.72
T3 [ref (T1)]	0.65	0.98	0.12	0.66	0.514	−1.32	2.61
T4 [ref (T1)]	0.19	1.25	0.02	0.15	0.881	−2.30	2.68
Lymph node yield	0.07	0.05	0.15	1.39	0.168	−0.03	0.16
Neoadjuvant therapy [ref (No)]	1.64	0.89	0.21	1.86	0.068	−0.12	3.41
Surgery approach [ref (Dixon)]	−1.92	1.16	−0.18	−1.66	0.101	−4.23	0.39
Operative time (min)	0.01	0.01	0.17	1.36	0.178	−0.00	0.02
Blood loss (mL)	0.01	0.00	0.18	1.42	0.159	−0.00	0.01

B, unstandardized regression coefficient; SE, standard error; β, standardized regression coefficient; CI, confidence interval; BMI, body mass index.

### Postoperative complications

The postoperative complication rate was 16.1% (5/31) in the Si group and 7.3% (4/55) in the Xi group. No significant differences were observed between the two groups in the frequencies of postoperative complications, including postoperative bleeding, abdominal infection, perineal infection, urinary system infection and anastomotic leakage (*p* > 0.05 for all; Table 6).

**Table 6:** Postoperative complications.

Variable	Si [n (%)]	Xi [n (%)]	*P*
Complications	5 (16.1%)	4 (7.3%)	0.198
Anastomotic leakage	1 (3.2%)	2 (3.6%)	1.000
Postoperative bleeding	2 (6.5%)	0 (0%)	0.127
Abdominal infection	1 (3.2%)	1 (1.8%)	1.000
Urinary tract infection	1 (3.2%)	0 (0%)	0.360
Perineal infection	0 (0%)	1 (1.8%)	1.000

### Perioperative short-term oncological outcomes

Pathological outcomes showed no significant differences for short-term oncological outcomes between two platforms (all *p* > 0.05). The Xi group had numerically higher rates of adequate DRM (100% vs. 93.5%, *p* = 0.130), CRM negativity (98.2% vs. 96.8%, *p* = 1.000), and complete mesorectal excision (96.4% vs. 90.3%, *p* = 0.608) compared to the Si group, though all comparisons were statistically non-significant (Table 7).

**Table 7:** Short-term oncological outcomes after surgery.

Variable	Si (*n* = 31)	Xi (*n* = 55)	P
DRM, cm			0.130
<1	2 (6.5%)	0 (0%)	
≥1	29 (93.5)	55 (100%)	
CRM			1.000
Negative	30 (96.8%)	54 (98.2%)	
Positive	1 (3.2%)	1 (1.8%)	
Mesorectal integrity			0.608
Grade 1	1 (3.2%)	1 (1.8%)	
Grade 2	2 (6.5%)	1 (1.8%)	
Grade 3	28 (90.3%)	53 (96.4%)	

DRM, distal resection margin; CRM, circumferential resection margin.

### Rate of anus preservation in rectal cancer within 5 cm of the anal verge

Table 8 presented comparable baseline characteristics for rectal cancer with ≤5 cm from the anal verge between the Si and Xi groups, including sex distribution (*p* = 0.704), BMI categories (*p* = 1.000), T stage (*p* = 0.530), and mean tumor distance from the anal verge (4.00 ± 0.38 vs. 4.29 ± 0.25 cm, *p* = 0.987). With balanced baseline characteristics between two groups, the Xi system demonstrated superior anus preservation rates (90.5% vs. 55.6%, *p* = 0.049).

**Table 8:** Comparison of anus preservation for low rectal cancer between the Si and Xi groups.

Variable	Si (*n* = 9)	Xi (*n* = 21)	*P*
Sex			0.704
Female	3 (33.3%)	9 (42.9%)	
Male	6 (66.7%)	12 (57.1%)	
BMI (kg/m^2^)			1.000
<18.5	1 (11.1%)	1 (4.8%)	
18.5–24.9	8 (88.9%)	19 (90.5%)	
>25	0 (0%)	1 (4.8%)	
T stage			0.530
T1	0 (0%)	3 (14.3%)	
T2	3 (33.3%)	8 (38.1%)	
T3	6 (66.7%)	10 (47.6%)	
T4	0 (0%)	0 (0%)	
Mean distance from the anal verge (cm) + SD	4.00 ± 0.38	4.29 ± 0.25	0.987
Anus preservation			0.049
Yes	5 (55.6%)	19 (90.5)	
No	4 (44.4%)	2 (9.5%)	

BMI, body mass index; SD, standard deviation.

### Long-term comparison of outcomes

**Figure 2:**
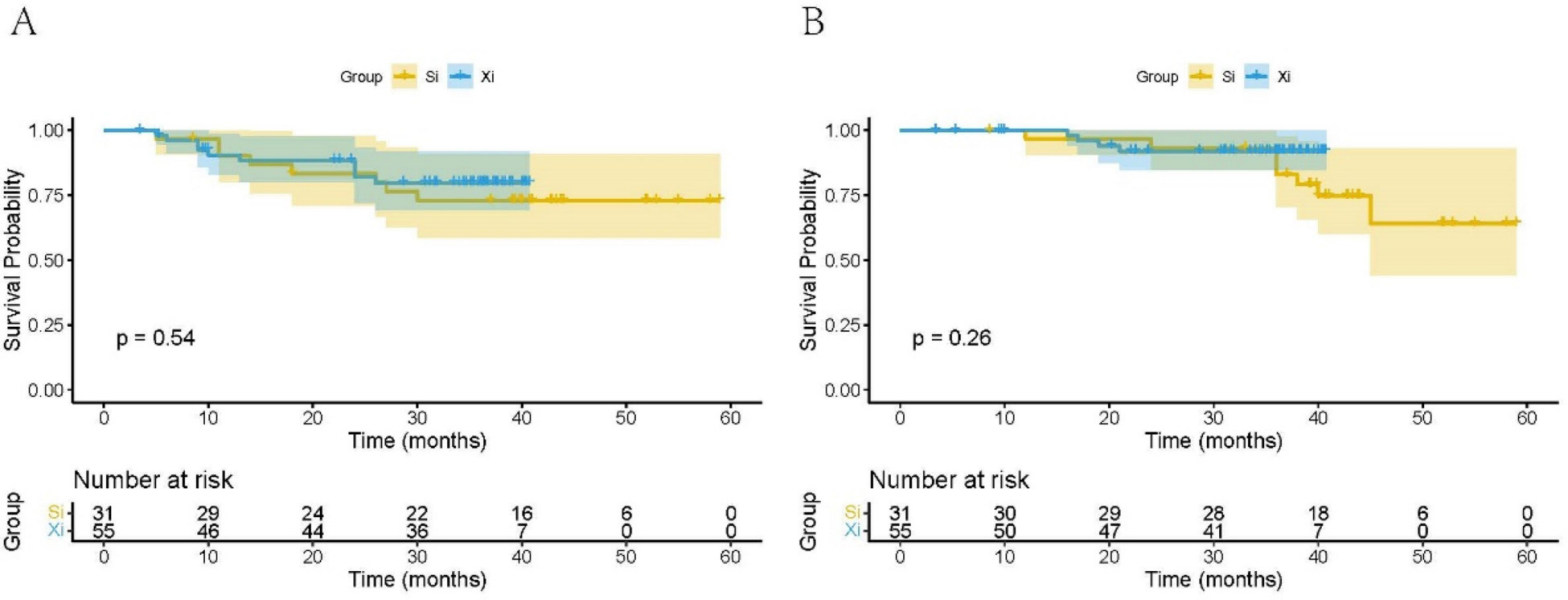
Kaplan–meier curves of DFS **(A)** and OS **(B)** for the Si and Xi robotic system groups. DFS, disease-free survival; OS, overall survival.

As shown in Figure 2, the Kaplan–Meier survival analysis was performed to evaluate the impact of the Si and Xi systems on prognosis in rectal resection patients, focusing on DFS and OS. In the Si group, the median follow-up for DFS and OS was 40 months (range 5–59 months) and 41 months (range 9–59 months), respectively. In the Xi group, the median follow-up times were 34 months (range 3–41 months) for DFS and 35 months (range 3–41 months) for OS. Table 9 showed the 1-year DFS rates for the Si and Xi groups were 90.1% vs. 90.5%, and the 3-year DFS rates for the Si and Xi groups were 73.0% vs. 79.8%. The 1-year OS rates for the Si and Xi groups were 96.7% and 98.0%, respectively, while the 3-year OS rates were 83.0% for the Si group and 92.0% for the Xi group, indicating a higher DFS or OS rate in the Xi group. However, it is important to note that no statistically significant differences were observed between the two groups in terms of DFS (*p* = 0.54) or OS (*p* = 0.26).

**Table 9:** Comparison of 1- and 3-year DFS and OS rates between Si and Xi surgical system .

Event	Group	1-year (%)	3-year (%)	95% CI	*P*
DFS	Si (*n* = 31)	90.1%	73.0%	41.15–54.59	0.54
	Xi (*n* = 55)	90.5%	79.8%	32.69–38.60	
OS	Si (*n* = 31)	96.7%	83.0%	45.66–55.78	0.26
	Xi (*n* = 55)	98.0%	92.0%	37.22–40.63	

SD, standard deviation; DFS, disease-free survival; OS, overall survival; CI, confidence interval.

In Cox regression analyses of DFS and OS, TNM stage was the only factor consistently associated with worse outcomes after adjustment (DFS: *p* = 0.041; OS: *p* = 0.029), with higher stages conferring greater risk relative to stage I. For DFS, neoadjuvant therapy remained independently protective (*p* = 0.002), whereas tumor deposit lost significance after adjustment despite a univariate association (*p* = 0.025). For OS, neoadjuvant therapy showed a protective effect in univariate analysis (*p* = 0.019) that attenuated in the multivariable model (*p* = 0.165). The da Vinci system (Xi vs. Si) was not associated with DFS (HR = 1.33, 95% CI: 0.53–3.37; *p* = 0.548) or OS (HR = 1.43, 95% CI: 0.76–2.67; *p* = 0.267) on univariate analysis and did not emerge as an independent predictor; likewise, age and sex were not significant for either endpoint (all *p* > 0.70; Tables 10, 11).

**Table 10:** Univariate and multivariate Cox regression analysis of DFS for patients with rectal cancer.

Variable	Univariate		Multivariate	
	HR (95% CI)	*P*	HR (95% CI)	*P*
da Vinci system		0.548		
Si (31)	Ref.			
Xi (55)	1.33 (0.53–3.37)			
Age (year-old)		0.893		
<60 (47)	Ref.			
≥60 (39)	0.94 (0.37–2.38)			
Sex		0.904		
Female (40)	Ref.			
Male (46)	1.06 (0.42–2.68)			
TNM stage		0.013		0.041
I (26)	Ref.		Ref.	
II (28)	1.38 (0.31–6.17)		1.58 (0.35–7.11)	
III (30)	3.83 (1.05–13.95)		3.52 (0.97–12.85)	
IV (2)	31.21 (2.70–361.35)		25.32 (1.99–322.63)	
Tumor deposit		0.025		0.495
No (71)	Ref.		Ref.	
Yes (15)	3.08 (1.15–8.22)		1.56 (0.44–5.57)	
Neoadjuvant therapy		< 0.001		0.002
No (75)	Ref.		Ref.	
Yes (11)	0.19 (0.07–0.49)		0.22 (0.08–0.58)	

DFS, disease-free survival; HR, hazard ratio; CI, confidence interval.

**Table 11:** Univariate and multivariate Cox regression analysis of OS for patients with rectal cancer.

Variable	Univariate		Multivariate	
	HR (95% CI)	*P*	HR (95% CI)	*P*
da Vinci system		0.267		
Si (31)	Ref.			
Xi (55)	1.43 (0.76–2.67)			
Age (year-old)		0.754		
<60 (47)	Ref.			
≥60 (39)	0.83 (0.26–2.63)			
Sex		0.995		
Female (40)	Ref.			
Male (46)	1.00 (0.57–1.77)			
TNM stage		0.004		0.029
I (26)	Ref.		Ref.	
II (28)	4.10 (0.42–39.87)		3.96 (0.41–38.44)	
III (30)	9.46 (1.16–77.57)		8.21 (0.99–68.06)	
IV (2)	383.41 (13.12–11,202.81)		168.53 (4.91–5,787.41)	
Tumor deposit		0.235		
No (71)	Ref.			
Yes (15)	2.22 (0.60–8.25)			
Neoadjuvant therapy		0.019		0.165
No (75)	Ref.		Ref.	
Yes (11)	0.25 (0.08–0.80)		0.40 (0.11–1.45)	

OS, overall survival; HR, hazard ratio; CI, confidence interval.

## Discussion

The Xi system has demonstrated significant improvements in design, functionality, and application scope compared to the Si system (13–15). This study validates the Xi system's technical advancements for rectal cancer surgery, demonstrating superior sphincter-preservation capability—particularly for low tumors (≤5 cm from the anal verge), emphasizing its clinical advantages over previous platforms. Key improvements include enhanced maneuverability through longer, more flexible arms ideal for the narrow pelvis, superior high-definition 3D visualization for deep pelvic dissection, and specialized instrumentation for precise low rectal resection. The system's ergonomic design and automated features address the unique spatial challenges of low rectal surgery while improving surgical efficiency (16).

### Learning curve advantages

A key finding was the Xi system's significant reduction in docking time, reflecting its streamlined setup process with laser-guided port placement and automatic arm alignment. The significantly shorter overall operative time with the Xi system compared to the Si demonstrated its superior workflow efficiency, likely attributable to its streamlined docking process, enhanced instrument maneuverability, and reduced need for intraoperative repositioning. Notably, the improved outcomes in the Xi group may also reflect the surgical team's increasing familiarity with robotic systems over time. As surgeons accumulated experience with both platforms, their growing proficiency in utilizing the Xi system's advanced features, such as its improved multi-quadrant access and integrated table motion, likely contributed to the observed performance benefits. Morelli *et al*. reported that the Xi system reduced docking time by 6 min and total operative time by 33.8 min vs. the Si platform in robotic rectal cancer surgery, attributed to its improved multi-quadrant access capability (17).

### Perioperative efficiency

The clinically meaningful reductions in operative time, blood loss and length of hospital stay with the Xi system translate directly to patient benefits, including decreased anesthesia exposure and lower transfusion requirements. Robotic surgery has been found in some studies to reduce estimated blood loss in obese patients, potentially lowering the need for transfusions (18, 19). These advancements reduce operative stress on surgeons, optimize workflow, and minimize patient trauma, which were in agreement with studies emphasizing the Xi system's enhanced ergonomic design and capabilities for multiquadrant surgeries (20–22).

### Short-term oncological outcomes

Both systems demonstrated equivalent safety pathological outcomes for DRM, CRM and complete mesorectal excision. The marginally lower rates in the Xi system did not achieve statistical significance. This may be because both techniques share similar approaches in intraperitoneal access and major procedural steps. Research by Monsellato et al. reinforces these findings, highlighting that both systems provide a safe platform for complex oncological procedures (23). Although both systems achieved equivalent pathological safety in DRM status, the Xi platform showed a consistent trend toward more reliable margin preservation. This technical nuance may explain its superior performance in low rectal cancer, where our subgroup analysis confirmed significantly higher sphincter preservation rates with Xi.

### Long-term outcomes

Robotic surgery for low rectal cancer has demonstrated favorable long-term prognostic impacts when performed by experienced surgeons, as highlighted in the retrospective cohort study from Fujita Health University (24). The analyses of DFS and OS showed no statistically significant differences between the Si and Xi. While long-term oncological outcomes remain comparable between systems, the Xi system's perioperative advantages in efficiency and reduced hospitalization represent meaningful quality-of-care improvements that justify its adoption, particularly in high-volume centers where workflow optimization is crucial. These results were consistent with previous literature emphasizing that surgical proficiency and adherence to oncological principles were critical determinants of long-term prognosis (25, 26). The subtle trends in survival curves could reflect the Xi system's potential for more precise resections, improving oncological margins. However, these benefits may be diluted in small, single-center studies with limited statistical power. The findings underscore the need for larger, multicenter trials to validate whether these survival trends are clinically meaningful or artifacts of sampling variability.

### Strengths and limitations

This study has several strengths, including the use of a consistent surgical team across both platforms, minimizing operator-dependent variability, and the inclusion of consecutive cases over a defined period reflecting real-world clinical practice. However, certain limitations should be acknowledged. The retrospective design introduces potential selection bias, though this was mitigated by applying strict inclusion criteria. The single-center nature and modest sample size may limit generalizability, particularly for rare complications or subtle outcome differences. Additionally, while we accounted for the learning curve effect in our analyses, the progressive surgeon experience with robotic systems over the study period remains a potential confounding factor. These limitations highlight the need for prospective, multicenter studies with larger cohorts to validate our findings.

## Conclusion

This study demonstrates that the Xi platform offers superior perioperative performance compared to the Si system, including shorter operative time, reduced blood loss, and shorter hospitalization, without compromising oncological safety. These advantages, likely resulting from improved instrumentation and enhanced visualization, suggest that the Xi system represents a significant technological advancement in robotic rectal cancer surgery. Additionally, Xi may offer advantages in sphincter preservation for low rectal cancers, although this requires further confirmation. Future research should explore how these innovations contribute to surgical standardization, training reproducibility, and long-term outcomes across diverse patient populations.

## Data Availability

The raw data supporting the conclusions of this article will be made available by the authors, without undue reservation.
